# A Preliminary Assessment of *Amblyseius andersoni* (Chant) as a Potential Biocontrol Agent against Phytophagous Mites Occurring on Coniferous Plants

**DOI:** 10.3390/insects12080664

**Published:** 2021-07-21

**Authors:** Ewa Puchalska, Stanisław Kamil Zagrodzki, Marcin Kozak, Brian G. Rector, Anna Mauer

**Affiliations:** 1 Section of Applied Entomology, Department of Plant Protection, Institute of Horticultural Sciences, Warsaw University of Life Sciences—SGGW, Nowoursynowska 159, 02-787 Warsaw, Poland; stanislaw_zagrodzki@sggw.edu.pl (S.K.Z.); anna.mauer@protonmail.com (A.M.); 2 Department of Media, Journalism and Social Communication, University of Information Technology and Management in Rzeszów, Sucharskiego 2, 35-225 Rzeszów, Poland; nyggus@gmail.com; 3 USDA-ARS, Great Basin Rangelands Research Unit, 920 Valley Rd., Reno, NV 89512, USA; brian.rector@usda.gov

**Keywords:** biological control, Phytoseiidae, spider mites, Tenuipalpidae, life tables

## Abstract

**Simple Summary:**

*Amblyseius andersoni* (Chant) is a predatory mite frequently used as a biocontrol agent against phytophagous mites in greenhouses, orchards and vineyards. In Europe, it is an indigenous species, commonly found on various plants, including conifers. The present study examined whether *A. andersoni* can develop and reproduce while feeding on two key pests of ornamental coniferous plants, i.e., *Oligonychus ununguis* (Jacobi) and *Pentamerismus taxi* (Haller). *Pinus sylvestris* L. pollen was also tested as an alternative food source for the predator. Both prey species and pine pollen were suitable food sources for *A. andersoni*. Although higher values of population parameters were observed when the predator fed on mites compared to the pollen alternative, we conclude that pine pollen may provide adequate sustenance for *A. andersoni* populations when prey are absent. Based on our results and due to the fact that the predator was previously recorded as sympatric with *O. ununguis* and *P. taxi*, we consider it to be a promising biocontrol agent of these pests.

**Abstract:**

Development, survival and reproduction of *Ambyseius andersoni* (Chant), a predatory mite widely distributed in Europe, were assessed on different food items. These included two key pests of ornamental coniferous plants, i.e., *Oligonychus ununguis* (Jacobi) and *Pentamerismus taxi* (Haller) and pollen of *Pinus sylvestris* L. The rationale behind these experiments was to provide a preliminary assessment of the potential of *A. andersoni* as a biocontrol agent of the above phytophagous arthropods and evaluate pine pollen as an alternative food source for the predator. Under laboratory conditions (23 ± 0.5 °C, 70 ± 10% RH and 16L:8D) *A. andersoni* was able to feed, develop and reproduce on all tested diets. The shortest development time (egg to female) was obtained when the predator fed on *P. taxi* (mean = 5.12 d) and the longest was on pine pollen (mean = 6.55 d). The r_m_ value was significantly higher on both tested prey (0.166 on *P. taxi* and 0.160 on *O. ununguis*) than on pollen (0.139). Thus, we do not recommend pine pollen for mass rearing of *A. andersoni*; however, we conclude that pollen may provide sufficient sustenance for the predator population under field conditions when prey are absent. The potential of *A. andersoni* as a biocontrol agent of *O. ununguis* and *P. taxi* is discussed.

## 1. Introduction

Coniferous plants play an important role in both ecological and economic settings. They are especially beneficial in terms of sequestering carbon from the atmosphere. Fast growth and the wood’s properties make many species of conifers ideal for timber production. Many of these trees and shrubs are also valuable as ornamental plants and gaining popularity for use in urban landscapes. However, the utility of these plants is closely related to their aesthetic quality and this may be influenced by harmful arthropod species. One of them is *Pentamerismus taxi* (Haller) (Tenuipalpidae), a false spider mite belonging to the superfamily Tetranychoidea in the order Prostigmata. This flattened red mite is commonly found on many wild and ornamental plants of the genus *Taxus*, growing in Europe and Asia [1,2,3,4,5,6]. According Bondareva and Chumak [6] this species’ status as an invasive pest is growing due to climate change. Another member of the Tetranychoidea superfamily—*Oligonychus ununguis* (Jacobi), the spruce spider mite (Tetranychidae)—is becoming a major pest of coniferous plants in nurseries, botanical gardens, urban parks and on Christmas tree plantations [7,8]. This polyphagous herbivore occurs worldwide on a wide range of host plants, including *Cryptomeria* spp., *Abies* spp., *Thuja* spp., *Juniperus* spp., *Larix* spp., *Pinus* spp., *Picea* spp. and *Taxus* spp. [9,10,11,12,13,14,15]. Both pests contribute to a significant reduction in the decorative value and health of plants [6,16,17].

The last few years, we have seen an intensification of the problem associated with spruce spider mite and tenuipalpids appearing on ornamental coniferous plants. Small body size (making the pests difficult to detect), short life cycle, and high fecundity, as well as changing climate, have favored the development of high populations of these mites [6,18,19,20]. For years, most of the programs used to control pests in coniferous plant nurseries have been based on chemical methods [21]. However, acaricides designed to reduce mite populations on conifers are not sufficiently effective [22,23], as studies of the occurrence and distribution of *O. ununguis* have shown significant numbers of this pest, even in intensively managed nurseries [8]. As such, there is a need to develop new alternatives to chemical tactics against mite pests in ornamental coniferous plantations. One of these methods is biological control, in which natural enemies are released to keep pest populations at low density level.

Phytoseiid mites are currently among the best studied biological control agents and are used extensively in programs to manage many groups of phytophagous arthropods, e.g., spider mites, eriophyid mites, scale insects, whiteflies, thrips [24]. Their beneficial use in plant protection programs has been well documented in the scientific literature, e.g., [25,26,27]. Biological methods based on augmentative releases of phytoseiid mites in vegetable and fruit crops are well-developed but such solutions are still lacking in the case of ornamental conifers. Complex studies of the Phytoseiidae fauna of conifers conducted in United States of America and Poland have revealed an abundance of *Amblyseius andersoni* (Chant) across many examined plant species [8,28,29]. The co-occurrence of this predatory mite with spruce spider mite was noticed on ornamental coniferous plants in Polish nurseries [14] and with *P. taxi* on wild-growing yews [30]. As a naturally occurring species in both Europe and the United States, *A. andersoni* seems to be better adapted to the local environment than other, non-native phytoseiid species. One advantage of indigenous natural enemies may be biological synchrony with potential prey species [31]. According to Janssen and Sabelis [32] the potential of predatory mites as natural enemies can be preliminarily assessed by determining values of demographic parameters that they achieve when fed on targeted prey species. Therefore, it is fruitful to conduct this type of laboratory research for the case of *A. andersoni* feeding on *O. ununguis* and *P. taxi*.

The effectiveness of a natural enemy in protected crops is largely related to its persistence even when the prey is scarce or absent, as the ability to utilize available alternative food sources is a critical feature of prospective biocontrol agents [33]. One of the most important alternative food items of many phytoseiid mites is pollen [34]. Some predatory mites can express even higher reproductive fitness by feeding on pollen than preying on phytophagous arthropods, e.g., [35]. However, pollen from different plant species differs in its suitability as food by particular phytoseiid species [36,37,38]. According to Addison et al. [38] the plant family Pinaceae is an important pollen provider for generalist predatory mite species, especially in early spring when prey abundance is low. Hence, it is useful to evaluate the suitability of pine pollen as an alternative food source for prospective biological control agents of conifers. The suitability of pollen of various pine species as alternative food for predatory mites has been tested for several phytoseiid species [39,40,41,42], but not *A. andersoni*.

The objective of this study was to provide a preliminary evaluation of the potential utility of *A. andersoni* as a biological agent against *O. ununguis* and *P. taxi*. For this purpose, developmental, reproductive, and demographic parameters of the predatory mite were measured during its feeding on targeted prey species, as well on an alternative food source that is presumed to be abundant in the field when pest mites are scarce, viz. pine (*Pinus sylvestris* L.) pollen.

## 2. Materials and Methods

### 2.1. Plant Material

Plants of *Picea abies* (L.) H. Karst cv. Nidiformis (avg. diameter 40 cm) and *Taxus baccata* L. (avg. high 70 cm) were grown in plastic pots (30 cm dia., 40 cm ht.) filled with a peat substrate. Plants of each species were kept in separate walk-in plant growth rooms (25 ± 5 °C, 65 ± 10% RH and 16L:8D photoperiod) and were used for mass rearing of the pests. In some growth rooms, six plants of a given species were placed close to each other to allow mites to move freely among them. Conifers were watered on alternate days. Another room contained only uninfested plants of both species.

The pollen of *P. sylvestris* was collected from plants growing in Kabaty Forest (Warsaw, Poland). The pollen was dried and stored in a freezer (−18 °C). For the experiments, it was thawed and kept in a refrigerator at 4 °C for a maximum of one week.

### 2.2. Phytophagous Mite Rearing

A stock colony of *P. taxi* was established with individuals collected from *T. baccata* growing in several urban parks in Warsaw, Poland. *Oligonychus ununguis* specimens were obtained from *Picea glauca* (Moench) Voss cv. Conica plants and *P. abies* trees growing in the same parks. About 80–100 females of each mite species were placed separately on shoots of *T. baccata* or *P. abies* cv. Nidiformis detached from uninfested plants. Infested shoots (approx. 15 cm) were kept in plastic beakers containing water in an environmental chamber (Sanyo MLR-350) at 23 ± 0.5 °C, 70 ± 10% RH and 16L:8D photoperiod. After laying eggs on shoots, females were slide-mounted in Heinze PVA medium for phase-contrast microscopic examination [43]. Shoots bearing progeny of each test pest were kept under these conditions for 3–4 weeks. Infested shoots were then transferred to shrubs growing in plant growth rooms. Every three weeks, two new (not infested) plants were added to each room to maintain the culture.

### 2.3. Stock Colony of Amblyseius andersoni

Stock colonies of *A. andersoni* were established with specimens (Anderline Pro Bioline AgroSciences Ltd., Little Clacton, UK) obtained from Hortico S.A. Company (Wroclaw, Poland). Each of the three colonies were maintained in the laboratory of the Department of Applied Entomology (SGGW) in an environmental test chamber (23 ± 0.5 °C, 70 ± 10% RH and 16L:8D photoperiod). Using a fine paintbrush, the predators were placed in breeding containers (18 cm × 15 cm × 7 cm). In each container a tile of black plastic (15 cm × 12 cm) was resting on a damp sponge (16 cm × 13 cm × 4.5 cm). Wet tissue paper strips (1 cm wide) formed a barrier around the tile. On the tissue paper, a ring of insect glue (Vitax^®^ fruit tree grease) was laid as a barrier against predator escape. Several roof-shaped pieces of transparent plastic with black sewing threads underneath were placed on the tile as a shelter where the phytoseiids could deposit their eggs. In each colony, *A. andersoni* was fed with either pine pollen or different stages of *O. ununguis* or *P. taxi*. Mites were reared under these conditions for at least four generations before conducting the experiment.

### 2.4. Experimental Procedures

All experiments were conducted in environmental chambers (23 ± 0.5 °C, 70 ± 10% RH and 16L:8D photoperiod) in experimental units similar to those used for rearing the predator but the plastic plate in each container was divided into six sub-arenas of 4 cm × 5 cm, separated by moistened tissue paper (1 cm wide) and a strip of glue. A piece of a transparent plastic sheet folded in the shape of a tent was placed over each sub-arena as a shelter and oviposition site for *A. andersoni* females. In each unit, phytoseiids were fed with surplus amounts of a food item corresponding to that provided in the stock colony: (1) larvae and nymphs of *O. ununguis*; (2) larvae and nymphs of *P. taxi*; or (3) pine pollen. Pollen was replenished every day with a fine paintbrush, about 0.5 mg per day. Overall, 234 replications were made.

To determine the duration and survival of each life stage of *A. andersoni*, gravid females taken from particular stock colonies were placed individually in experimental units. After 12 h, one egg was left in each unit; the females and the remaining eggs were removed. Observations were made twice a day until all individuals reached adulthood. The presence of an exuvium indicated successful molting to the next developmental stage. After completing immature development, females were paired with males. When a male died or escaped, a new one was added from the respective stock colony. The experimental units were examined every 24 h to determine the duration periods of pre-oviposition, oviposition, post-oviposition, as well as longevity and fecundity of females. Eggs laid on a given day were placed in a single unit and reared to adulthood to determine the sex ratio of the progeny.

### 2.5. Data Analysis

Life tables were constructed from the observed age-specific survival rate (l_X_, percent of surviving females at the instant x) and age-specific fecundity rate (m_X_, number of female eggs laid per female per day). The net reproductive rate (R_0_), mean generation time (T), intrinsic rate of increase (r_m_), and finite rate of increase (λ) were calculated using the method recommended by Birch [44].

Because of nested observations within trays, we used linear mixed-effect modeling [45] to analyze the effect of diet on the traits studied. The fitted models were checked using graphical methods [45,46]; all of them showed good fit. To compare the diets in pairs, we used the corresponding linear hypotheses [47], with no adjustment for multiple testing [48]. The analyses were conducted using R [49], with the nlme [50] and multcomp [47] packages.

To compare the demographic parameters between the pairs of the foods, pair-wise comparisons of these parameters were applied using the jackknife method [51], without adjustment for multiple testing [52].

## 3. Results

Developmental time of eggs, larvae and protonymphs of *A. andersoni* was significantly affected by the diet (Table 1). Eggs deposited by females fed with *O. ununguis* or *P. taxi* hatched after 1.53 and 1.48 days (respectively), whereas those laid by females reared on pine pollen hatched significantly later (1.79 days) (*p* = 0.03). The larval stage was the shortest one, ranging from 0.62 days on *P. taxi* to 0.84 days on pine pollen (*p* < 0.001). Additionally, protonymphal stage duration was shortest on *P. taxi* (1.40 days) and longest while feeding on pine pollen (2.09 days) (*p* < 0.001). No significant difference in deutonymph development time was observed (*p* = 0.228) (Table 1).

On both tested prey species, as well as on pine pollen, *A. andersoni* reached maturity, but the egg to adult period of both sexes was significantly influenced by the diet (*p* < 0.001) (Table 1). Mean developmental time of females and males was longer on pine pollen than on prey species. Female development was shortest during feeding on *P. taxi* (5.12 days), whereas mean developmental time of males was similar on both the prey species (5.21 days on *P. taxi* and 5.47 days on *O. ununguis*) (Table 1). Food source also affected *A. andersoni* immature survival. While feeding on *P. taxi*, over 90% of predator specimens reached maturity, 87.23% on *O. ununguis*, and the lowest survival was noted on pine pollen (79.25%), which was a consequence of relatively high proto- and deutonymph mortality (Table 1).

The pre-oviposition, reproductive, and post-reproductive periods, as well as total fecundity and longevity of *A. andersoni* females were significantly dependent on the diet fed to the predator (Table 2). Time to first egg deposition was similar on *O. ununguis* and pine pollen (mean 3.28 and 3.50 days), and shortest when females preyed on *P. taxi* (mean 2.02 days). The mean reproductive period (oviposition) of *A. andersoni* was longer on *P. taxi* (mean 24.4 days) compared to *O. ununguis* (mean 20.4 days) and pine pollen (mean 19.1 days). On all food items *A. andersoni* females laid eggs almost to the end of their lives; the post-oviposition period ranged from 1.47 days to 2.7 days. The total number of eggs deposited by females reared on *O. ununguis* (mean 29.2 eggs/female) and *P. taxi* (mean 33.3 eggs/female) was significantly higher than that of females maintained on pine pollen (mean 24.2 eggs/female) but daily fecundity (oviposition rate) of *A. andersoni* was not affected by diet. The longest female lifespan was registered with *A. andersoni* fed on *P. taxi* (mean 28.3 days) and the shortest on pine pollen (mean 23.1 days) (Table 2).

**Figure 1:**
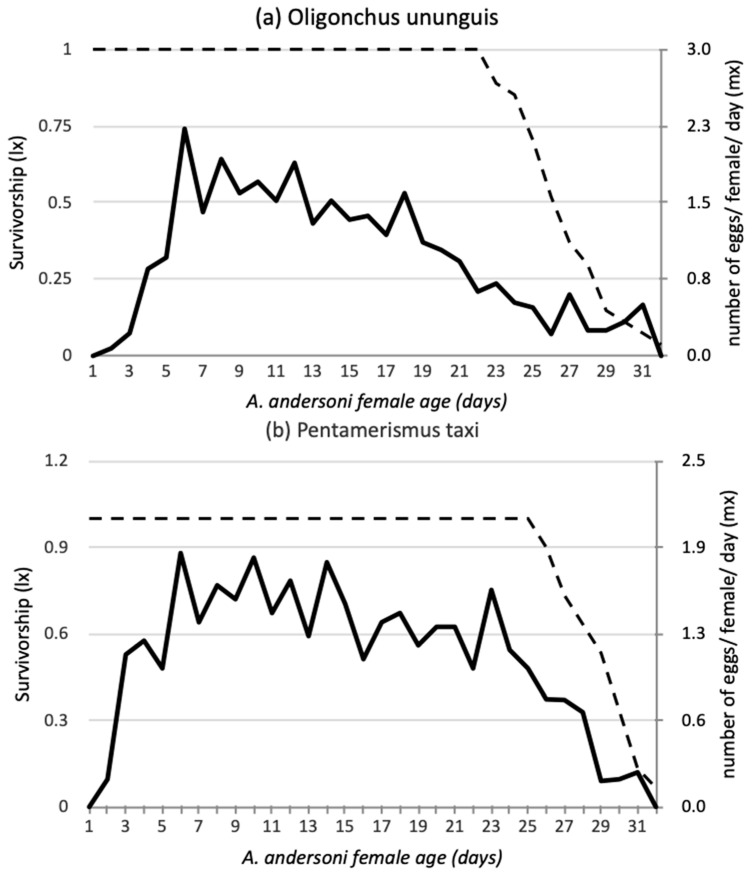

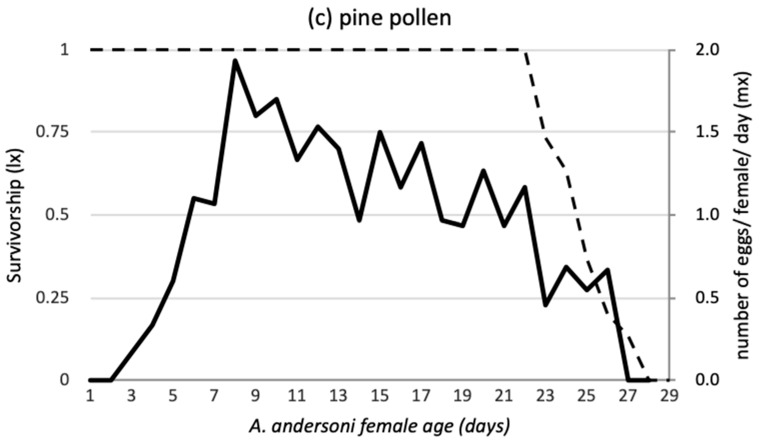
Age-specific survival rate (m_x_-dashed line) and fecundity (l_x_-solid line) of *Amblyseius andersoni* females reared on (**a**) *Oligonychus ununguis,* (**b**) *Pentamerismus taxi* and (**c**) *Pinus sylvestris* pollen.

Age-specific survival rate and fecundity of *A. andersoni* females (Figure 1) were used to calculate demographic parameters of the predatory mite feeding on different diets (Table 3). *Amblyseius andersoni* fed on pine pollen showed the lowest intrinsic rate of increase (r_m_ = 0.139) and finite rate of population increase (λ = 1.15). These values were significantly different from those obtained when the predator fed on either prey species. The predator population reared on pine pollen also had the longest mean generation time (T = 19.7) and the lowest net reproductive rate (R_0_ = 15.4) (Table 3).

## 4. Discussion

The ability of some phytoseiid mites, e.g., *Typhlodromus americanus* Chant and Yoshida-Shaul, *Galendromus* (*Galendromus*) *annectens* (De Leon), *G.* (*G.*) *floridanus* (Muma) (junior synonym *G. helveolus*), *G.* (*G.*) *occidentalis* (Nesbitt), and *Neoseiulus fallacis* (*Garman*), to suppress spruce spider mite populations has been the subject of several laboratory and field studies [21,53,54,55,56]. Some of these surveys have shown that two of the aforementioned predators, *G.* (*G.*) *occidentalis* and *N. fallacis*, can successfully reduce populations of the pest [21,53]. However, geographical distributions of both species were mainly reported in North and South America [57] with incidental occurrence in Europe where *O. ununguis* is a key pest of ornamental coniferous plants [8,58]. In Europe, *G.* (*G.*) *occidentalis* was noted only in Austria [59], whereas *N. fallacis* has been recorded in Germany [60] and in Poland [61,62]. By contrast, *A. andersoni* is widespread in Europe [57] and may overwinter on trees and in ground litter [63,64]. According to McMurtry et al. [65], *A. andersoni* has been classified as a type III-b among Phytoseiidae lifestyles; species from this category are considered to be generalist predators living on glabrous leaves of deciduous plants, but *A. andersoni* was recorded many times on different species of coniferous plants, e.g., [29,66,67,68], often sympatric with *O. ununguis* [30,69]. Generalist predators of type III can feed and reproduce on wide range of supplied food, including mite prey in many families of the suborder Prostigmata, among others, e.g., Tetranychiade and Tenuipalpidae [65]. Our results also show that *A. andersoni* is able to complete its life cycle and successfully reproduce on representatives of these latter families, viz., *O. ununguis* (Tetranychiade) and *P. taxi* (Tenuipalpidae).

According to Lehman [13], describing an integrated approach to *O. ununguis* management, the reproductive rate of phytoseiid mites is usually somewhat lower than that of the spider mites constituting their prey, causing predator populations to lag behind rapidly growing spruce spider mite populations, and as a consequence may allow significant damage to occur before the pest population is in check. Akita [70] showed that total fecundity of *O. ununguis* reared on fir (*Abies fraseri* (Pursh) Poir.) was as high as 43 eggs/female, which was almost 1.5 times greater than the fertility of *A. andersoni* preying on *O. ununguis* (see Table 2). However, the aforementioned study was conducted at 25 °C, whereas at 23 °C reproductive parameters of the predator and the prey (feeding on fir) were comparable (Table 2 of this study; [19]).

Some authors suggest the use of empirical r_m_ values as a useful indicator for selecting promising biocontrol candidates [71,72,73]. In theory, a predator that has a population growth rate equal to or greater than its prey should efficiently regulate the population of its prey [73,74]. The results presented here show that the value of the intrinsic rate of population increase (r_m_) of *A. andersoni* feeding on *O. ununguis* (r_m_ = 0.160) was much higher than that of *O. unuguis* feeding on fir (r_m_ = 0.125) or on spruce (r_m_ = 0.108) [19,75]. This is partly because *A. andersoni* feeding on *O. ununguis*, is able to reach maturity three times faster than its prey. The development time of *O. ununguis* on fir was 17.38 days [19] and of spruce 15.3 days [75]. The high values of developmental and population parameters of *A. andersoni*, observed during its feeding on *O. ununguis*, suggest that this prey is a nutritious food for the predator. Moreover, given that predator–prey population dynamics depend on the relative r_m_ of the protagonists [32], we hypothesize that *A. andersoni* has great potential as a natural enemy of *O. ununguis*.

In our surveys, the highest values of reproductive and population parameters were obtained for *A. andersoni* feeding on the other tested prey—*P. taxi*. Unfortunately, we cannot compare these parameters between the predator and the prey as there is no available data on the developmental, reproductive or population parameters of *P. taxi*. We can, however, compare values of the parameters achieved by *A. andersoni* feeding on *P. taxi* with those estimated on other prey species that have been studied as targets of this predator. *Amblyseius andersoni* has been evaluated as a biocontrol agent against spider mites occurring in vineyards [76,77,78], and orchards [64,79,80,81]. Amano and Chant [82] studied its developmental and reproductive parameters on *Tetranychus pacificus* McGregor, one of the most important spider mite pests in California vineyards [83]. Development time of both females and males of *A. andersoni* feeding on *T. pacificus* was about two days longer than on *P. taxi*, but total fecundity was higher on *T. pacificus* (46.33 eggs/female) than on *P. taxi* (33.3 eggs/female) ([82] vs. Table 2 of this study). Some authors mention *A. andersoni* as an effective natural enemy of another spider mite species, *Panonychus ulmi* Koch [79,84,85]. Comparing our findings with those of Lorenzon et al. [35], *A. andersoni* females feeding on *P. taxi* laid 1.5 times more eggs (33.3 eggs/female) than when preying on *P. ulmi* (22.2 eggs/female) and reached adulthood in a comparable or shorter time (5.12 days on *P. taxi* and 5.5 days on *P. ulmi*). Moreover, survival of *A. andersoni* (percentage of specimens reaching maturity) was higher on *P. taxi* (90.91%) than on *P. ulmi* (84%). Nevertheless, the r_m_ value of the predator population estimated by Lorenzon et al. [35] on *P. ulmi* (0.175) was higher than that on *P. taxi* (0.166), which may result from the longer lifespan of females feeding on *P. ulmi* (over 40 days) than on *P. taxi* (28.3 days). It is worth noting that Lorenzon et al. [35] conducted tests at a slightly higher temperature (24 °C) than in this study (23 °C) and many studies have shown that temperature significantly affects the life history of phytoseiid mites [86,87,88,89]. On the basis of the above comparison, we hypothesize that *P. taxi* is as valuable as prey for *A. andersoni* as the aforementioned species of spider mite pests of orchards or vineyards.

One of the objectives of the current research was the evaluation of pine pollen as an alternative food for *A. andersoni*. Although some phytoseiid mite species (e.g., *Euseius rubicolus* (van der Merwe and Ryke) = *Euseius addoensis* (McMurtry), *Iphiseius degenerans* (Berlese), *Neoseiulus cucumeris* (Oudemans)) could not exploit pollen of *P. sylvestris* as a food source and suffered from high pre-imaginal mortality [90,91], this pollen was suitable for *A. andersoni*. The development time of the predator on pine pollen was longer and the total fecundity was lower than on both examined prey species, but this food source sustained growth of *A. andersoni* populations. It can be assumed that pine pollen settling on needles and shoots of conifers in spring may favor early season development of *A. andersoni* populations and allow the predator to survive periodic shortages of other food. It is possible that *A. andersoni* could utilize pollen of other conifer species on which this predatory mite occurs (e.g., spruces, firs) but there is no such data in the literature. With the exception of pines [39,91,92] and Douglas fir [54], pollen from other conifers has not been tested as a supplementary food source for phytoseiid mites. *Typhlodromus americanus* Chant and Yoshida-Shaul fed with Douglas fir pollen did not reach adulthood [54]. Studies on suitability of this and other conifer pollens as sustenance for *A. andersoni* would improve our knowledge about the range of alternative food items that the predator can find on coniferous plants. However, pines typically produce great amounts of pollen, easily transported by wind [93,94], thus it may be available for phytoseiid mites not only on pines but also on other plant species on which it settles [38,95].

The possibility of laboratory rearing of phytoseiid mites on pollens of various plants was confirmed in many studies, e.g., [96,97,98,99]. However, it seems that *P. sylvestris* pollen is not suitable for mass rearing of *A. andersoni*, as higher development and reproductive parameters of this predator were recorded during its rearing on *Typha latifolia* L. pollen [35] or *Mesembryanthemum criniflorum* L. pollen [100].

A compelling case in favor of further work on *A. andersoni* as a biocontrol agent of phytophagous mites occurring on ornamental coniferous plants is the fact that this species of predatory mite is an existing commercial product used worldwide in many greenhouses or field programs (patent number PL/EP 2048941). This means there is no need to re-develop the mass breeding process for this species. Moreover, according to Duso et al. [78], the presence of many phytoseiid species in crops is limited by their susceptibility to pesticides while *A. andersoni* is one of the dominant Phytoseiidae species in commercial vineyards and orchards [78,101] due to its tolerance or resistance to some pesticides [27,78,102,103]. This opens the possibility of using it in integrated pest management conducted in ornamental nurseries. Due to the high potential of *A. andersoni* as a natural enemy of *O. ununguis* and *P. taxi*, future studies assessing the feasibility of the predator’s augmentative release against these pests in the outdoor production of coniferous plants are warranted.

## 5. Conclusions

Based on the data presented here, *Amblyseius andersoni* is a promising candidate for biocontrol of two key pests of coniferous plants, *O. ununguis* and *P. taxi*. This predatory mite is able to prey, develop and reproduce on both tested pests. High values of reproductive and population parameters achieved by *A. andersoni* while preying on *O. ununguis* and *P. taxi* may indicate that these phytophagous arthropods are a nutritious food source for the predator. *Pinus sylvestris* pollen can be classified as an alternative food source for *A. andersoni*. Although the values of population parameters of *A. andersoni* were higher on the tested prey species, the predator reached maturity and oviposited while feeding only on pine pollen.

## Figures and Tables

**Table 1:** Effect of different diets on the development and survival rate of *A. andersoni.* Values with different letters in a row differ significantly at *p* ≤ 0.05.

Developmental Stages	Diet Item			Calculated *p*-Value
	*O. ununguis*	*P. taxi*	Pine Pollen	
**Development (days ± SE)**				
Egg	1.53 ± 0.08 a	1.48 ± 0.07 a	1.79 ± 0.07 b	0.03
Larva	0.76 ± 0.04 b	0.62 ± 0.03 a	0.84 ± 0.04 b	<0.001
Protonymph	1.70 ± 0.09 b	1.40 ± 0.06 a	2.09 ± 0.07 c	<0.001
Deutonymph	1.61 ± 0.08	1.66 ± 0.07	1.79 ± 0.07	0.228
Egg to female	5.69 ± 0.13 b	5.12 ± 0.01 a	6.66 ± 0.16 c	<0.001
Egg to male	5.47 ± 0.13 a	5.21 ± 0.14 a	6.36 ± 0.20 b	<0.001
Egg to adult	5.61 ± 0.01 b	5.15 ± 0.08 a	6.55 ± 0.14 c	<0.001
**Survival (%)**				
Egg	95.74	96.36	98.11	
Larva	95.56	97.67	96.15	
Protonymph	95.35	97.62	92.00	
Deutonymph	100	97.56	91.30	
Percentage reaching maturity	87.23	90.91	79.25	

**Table 2:** *Amblyseius andersoni* female reproductive parameters and longevity (mean ± SE). Values with different letters in a column differ significantly at *p* ≤ 0.05.

Diet Item	Total Fecundity (Eggs/Female)	Oviposition Rate (Eggs/Female/Day)	Longevity (Days)	Preoviposition (Days)	Oviposition (Days)	Postoviposition (Days)
*O. ununguis*	29.2 ± 1.19 b	1.09 ± 0.03	26.3 ± 0.51 b	3.28 ± 0.22 b	20.4 ± 0.82 a	2.70 ± 0.28 b
*P. taxi*	33.3 ± 0.48 b	1.18 ± 0.01	28.3 ± 0.38 c	2.02 ± 0.11 a	24.4 ± 0.41 b	1.83 ± 0.25 a
Pine pollen	24.2 ± 0.54 a	1.05 ± 0.02	23.1 ± 0.31 a	3.50 ± 0.17 b	19.1 ± 0.30 a	1.47 ± 0.19 a
*p*-value	0.002	0.117	<0.001	<0.001	<0.001	0.001

**Table 3:** Mean (±SE) life table parameters of *Amblyseius andersoni* reared on *Oligonychus ununguis*, *Pentamerismus taxi* or pine pollen. Values with different letters in a row differ significantly at *p* ≤ 0.05.

	*Oligonychus ununguis*	*Pentamerismus taxi*	Pine Pollen
R_0_ (net reproductive rate)	18.7 ± 0.79 b	23.3 ± 0.34 c	15.4 ± 0.34 a
T (mean generation time)	18.3 ± 0.22 a	19.0 ±0.17 b	19.7 ± 0.18 c
r_m_ (intrinsic rate of population increase)	0.160 ± 0.002 b	0.166 ± 0.001b	0.139 ± 0.001 a
λ (finite rate of population increase)	1.17 ± 0.002 b	1.18 ± 0.001 c	1.15 ± 0.001 a

## Data Availability

Not applicable.
